# Randomized crossover trial comparing cervical spine motion during tracheal intubation with a Macintosh laryngoscope versus a C-MAC D-blade videolaryngoscope in a simulated immobilized cervical spine

**DOI:** 10.1186/s12871-020-01118-3

**Published:** 2020-08-15

**Authors:** Hyesun Paik, Hee-Pyoung Park

**Affiliations:** 1 Department of Anesthesiology and Pain Medicine, Armed Forces Capital Hospital, 81, Saemaul-ro 177 gil, Bundang-gu, Seongnam, 13590 South Korea; 2 Department of Anesthesiology and Pain Medicine, Seoul National University Hospital, Seoul National University College of Medicine, 101, Daehak-ro, Jongno-gu, Seoul, 03080 South Korea

**Keywords:** Cervical spine motion, Intubation, Macintosh laryngoscope, C-MAC D-blade videolaryngoscope, Cervical immobilization

## Abstract

**Background:**

Maintaining cervical immobilization is essential during tracheal intubation in patients with unstable cervical spines. When using the Macintosh laryngoscope for intubation in patients with cervical immobilization, substantial neck extension is required for visualization of the glottis. However, the C-MAC D-Blade videolaryngoscope may require less neck extension due to its acute angulation. We hypothesized that C-MAC D-Blade videolaryngoscopic intubation would result in less cervical spine movement than Macintosh laryngoscopic intubation. We compared the effects of C-MAC D-Blade videolaryngoscopic intubation and Macintosh laryngoscopic intubation in terms of cervical spine motion during intubation in patients with simulated cervical immobilization.

**Methods:**

In this randomized crossover study, the cervical spine angle was measured at the occiput–C1, C1–C2, and C2–C5 segments before and during tracheal intubation with either a C-MAC D-Blade videolaryngoscope or Macintosh laryngoscope in 20 patients, with application of a neck collar for simulated cervical immobilization. Cervical spine motion was defined as the change in angle measured before and during tracheal intubation.

**Results:**

The cervical spine motion at the occiput–C1 segment was measured at 12.1 ± 4.2° and 6.8 ± 5.0° during Macintosh laryngoscopic and C-MAC D-blade videolaryngoscopic intubation, respectively, corresponding to a 44% reduction in cervical spine motion when using the latter device (mean difference, − 5.3; 98.33% CI: − 8.8 to − 1.8; *p* = 0.001). However, there was no significant difference between the two intubation devices at the C1–C2 segment (− 0.6; 98.33% CI: − 3.4 to 2.2; *p* = 0.639) or C2–C5 segment (0.2; 98.33% CI: − 6.0 to 6.4; *p* = 0.929).

**Conclusions:**

The C-MAC D-Blade videolaryngoscope causes less upper cervical spine motion than the Macintosh laryngoscope during tracheal intubation of patients with simulated cervical immobilization.

**Trial registration:**

This study was registered at ClinicalTrials.gov on June 26, 2018 (NCT03567902)**.**

## Background

In general, when the Macintosh laryngoscope is used for tracheal intubation, extension of the head and flexion of the lower cervical spine are required to achieve alignment of oral, pharyngeal, and laryngeal axes. However, alignment of these three airway axes during Macintosh laryngoscopic intubation can cause adverse events, such as secondary damage of the spinal cord, in patients with an unstable cervical spine [1, 2]. Cervical spine immobilization via manual in-line stabilization of the neck or application of a cervical collar during intubation is necessary in cases with unstable cervical spines to avoid secondary neurologic injuries, which may result from excessive neck extension during Macintosh laryngoscopic intubation. Numerous studies have shown that Macintosh laryngoscopic intubation can cause substantial cervical spine movement, particularly upper cervical extension in patients with neck immobilization. Alternative intubation devices, for example videolaryngoscopes, lighted stylets, and intubating laryngeal masks, have been used to decrease cervical spine motion [3–8].

In clinical practice, various videolaryngoscopes have been used to prevent an unexpected failure during intubation in patients with unstable cervical spines [9–11]. The C-MAC D-Blade videolaryngoscope (Karl Storz, Tuttlingen, Germany) has a highly angulated blade tip, which provides easy intubation of patients with difficult airways [12, 13]. Unlike Macintosh laryngoscopic intubation, C-MAC D-Blade videolaryngoscopic intubation does not necessitate extreme neck extension for alignment of the three airway axes to visualize the glottis. Although a recent study demonstrated the superiority of the C-MAC D-Blade videolaryngoscope over the Macintosh laryngoscope in terms of cervical motion and glottis visualization, [14] the effect on cervical spine motion of C-MAC D-Blade videolaryngoscopy has not been investigated extensively.

The purpose of this study was to compare cervical spine motion between C-MAC D-Blade videolaryngoscopy and Mackintosh laryngoscopy during tracheal intubation in patients with simulated cervical immobilization. We hypothesized that the former device would be associated with less cervical spine motion.

## Methods

### Patient population

Ethical approval for this study (No.: 1804–123-940) was provided by the Institutional Review Board of Seoul National University Hospital, Seoul, Korea on June 4, 2018. This study was conducted under Good Clinical Practice Guidelines and adhered to the applicable Consolidated Standards of Reporting Trials (CONSORT) guidelines. We obtained written informed consents from all patients. This study was registered prior to patient enrollment at (NCT03567902, Principal investigator: Hee-Pyoung Park, Date of registration: June 26, 2018).

Twenty adult patients undergoing an elective endovascular cerebral aneurysm coiling under general anesthesia in July 2018 were enrolled. The exclusion criteria were as follows: a history of upper airway surgery due to polyp, tumor, inflammation, or trauma; risk factors for aspiration, such as gastroesophageal reflux disease or gastrointestinal obstruction; coagulopathy; body mass index > 30 kg/m^2^; and a history of cervical spine disease.

### Randomization

**Fig. 1:**
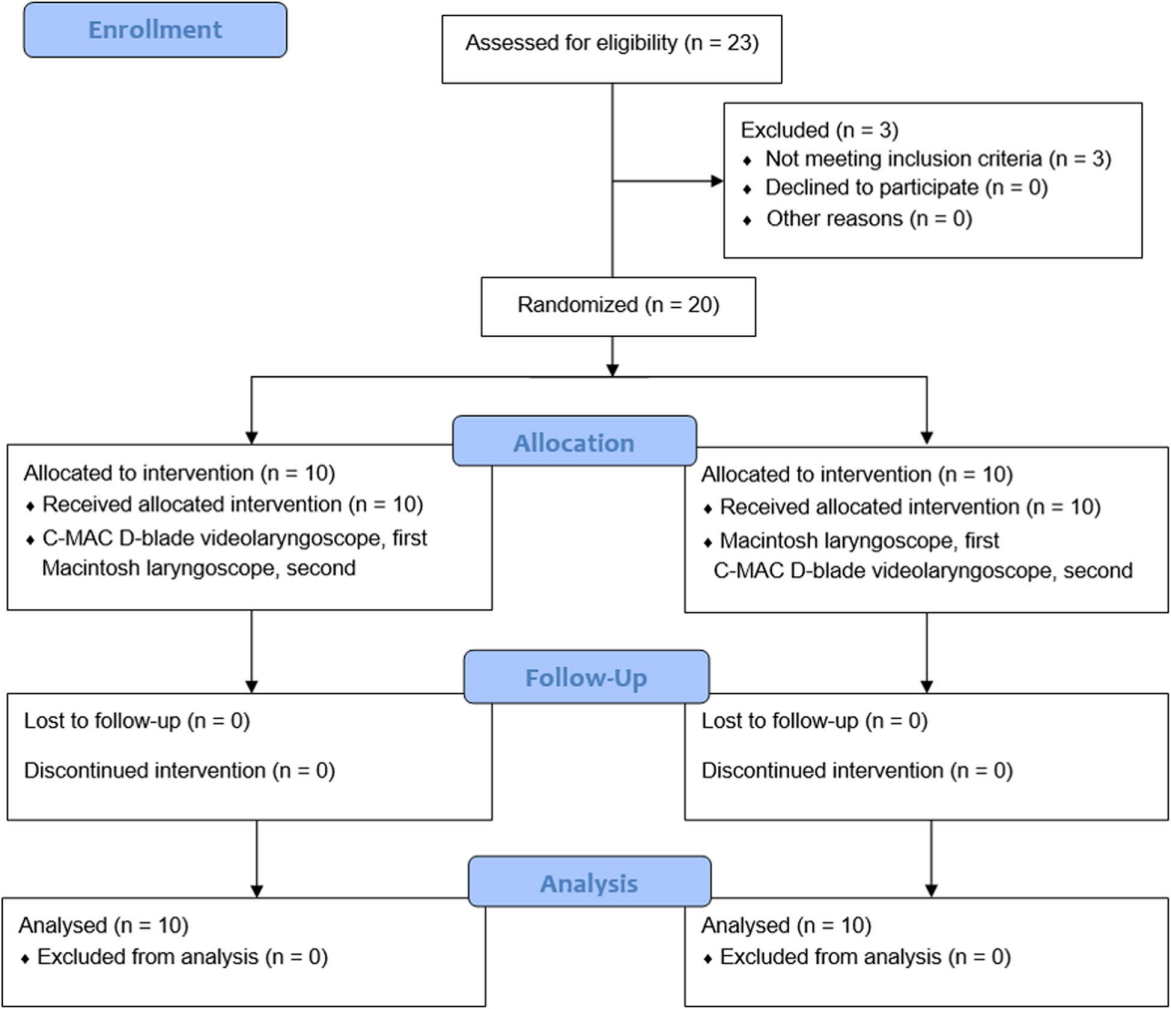
CONSORT flow diagram

Each patient experienced two consecutive tracheal intubations with a Macintosh laryngoscope (blade #3 for females, blade #4 for males) and a C-MAC D-Blade videolaryngoscope. We randomized patients to one of two groups depending on the intubation device used for the first tracheal intubation (Fig. 1): the Macintosh laryngoscope first group (*n* = 10) or C-MAC D-Blade videolaryngoscope first group (n = 10)**.** In the Macintosh laryngoscope first group, the first tracheal intubation was carried out with a Macintosh laryngoscope, and the second with a C-MAC D-Blade videolaryngoscope. In the C-MAC D-Blade videolaryngoscope first group, tracheal intubation using the two devices was performed in the reverse order.

Group allocation was performed in a 1:1 ratio by a blinded observer according to a randomization code using a computer program and concealed in sealed envelopes and revealed before anesthesia induction.

### Study protocol

Except the intubation sequence, other all procedures were identical in both groups. All intubations were performed by one skilled anesthesiologist. Patients entered the angiography room without premedication and underwent non-invasive blood pressure measurement, pulse oximetry, three-channel electrocardiography and bispectral index monitoring (A-2000 XP; Aspect Medical Systems, Newton, MA, USA). To evaluate the airway, the Mallampati score, thyromental distance, inter-incisor gap, sternomental distance, and neck circumference were recorded with the patient in the sitting position. Anesthesia was induced and maintained by propofol and remifentanil using a TCI system (Orchestra® Base Primea; Fresenius Kabi, Bad Homburg, Germany). To facilitate tracheal intubation, rocuronium (0.6–0.8 mg/kg) was administered.

After a patient was anesthetized, we simulated cervical spine immobilization by application of a cervical collar (Philadelphia® Tracheotomy Collar; Össur, Reykjavik, Iceland). The patient’s head was placed on a head rest of 5 cm height with a neutral position. A baseline sagittal image of the cervical spine was obtained using a biplanar angiographic unit (Integris Allura; Philips Medical Systems, Best, The Netherlands).

In the C-MAC D-Blade videolaryngoscope first group, using a C-MAC D-Blade videolaryngoscope, the patient’s trachea was intubated with a polyvinylchloride tracheal tube (Mallinckrodt™ Endotracheal Tube; Medtronic, Minneapolis, MN, USA), with an internal diameter of 7.0 mm for females and 7.5 mm for males. A malleable stylet was threaded through the endotracheal tube and its distal tip was angulated anteriorly by approximately 60 degrees. A jaw-thrust maneuver was not performed during intubation. When the epiglottis and vocal cords were obviously showed on the monitor, the endotracheal tube was advanced until its distal end was placed just before the vocal cords; it was then smoothly retracted out of the oral cavity. The intubation time was measured as the interval between insertion of the intubating device by incisor and just before passage of the endotracheal tube into the vocal cords. When the distal end of the endotracheal tube was positioned just before the vocal cords, the first sagittal radiographic image of the cervical spine during C-MAC D-blade videolaryngoscopic intubation was taken. After the patient’s head had been repositioned to neutral, the second baseline sagittal image of the cervical spine was obtained prior to the second intubation attempt, in which a Macintosh laryngoscope with a #3 or #4 blade was used. As with the first intubation attempt, the malleable stylet was threaded through the endotracheal tube and its distal tip was angulated anteriorly by approximately 60 degrees. A jaw-thrust maneuver was not performed. The blade of the Macintosh laryngoscope was then lifted to show the epiglottis and vocal cords, and the view was checked by the anesthesiologist. When the distal end of the endotracheal tube was positioned just before the vocal cords, a second sagittal image of the cervical spine during Macintosh laryngoscopic intubation was obtained. Then, the trachea was intubated. In the Macintosh laryngoscope first group, all procedures were identical aside from the intubating device sequence.

The intubation time in each attempt was limited to 90 s. For patient safety, when the intubation time reached the time limit, or if the pulse oximetry measurement detected to < 90%, mask ventilation was performed for 2 min and after that intubation reattempted. A total of three intubation attempts were permitted in each patient. Failed intubation was determined as failure of tracheal intubation in spite of three consecutive attempts. In case of failed intubation, an alternative intubation device, such as lighted stylets, intubating laryngeal masks or fiberoptic bronchoscope, was used at the anesthesiologist’s discretion. All patients were extubated after completing the endovascular intervention procedures and transferred to the post-anesthesia care unit.

### Study end points

**Fig. 2:**
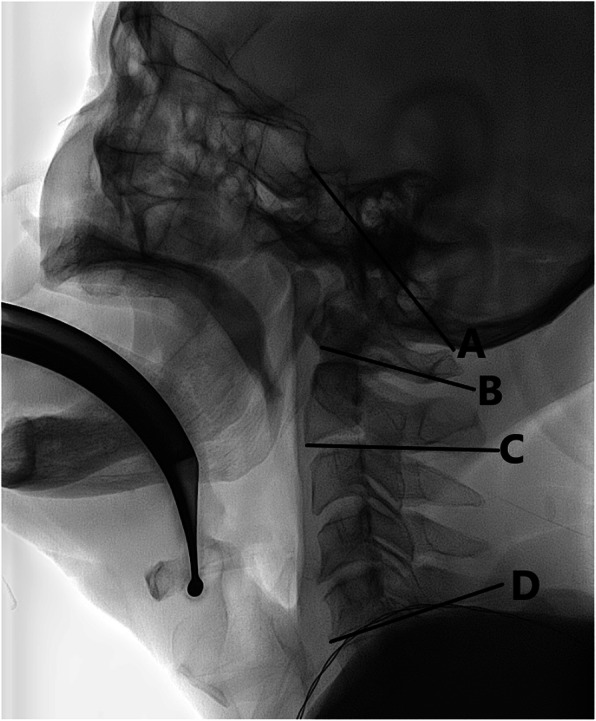
Vertebral reference lines. The reference line for the occiput was defined as a line between the base of the sella and the opisthion (line **a**), and the C1 reference line as a line between the lower cortical margin of the anterior arch of C1 and the lower cortical margin of the C1 spinous process (line **b**). The C2 reference line was defined as a line between the anterior, inferior margin of the C2 body and the lower cortical margin of the C2 spinous process (line **c**). The C5 reference line was a tangent along the superior end-plate of the C5 vertebral body (line **d**)

The primary outcome measure was the amount of cervical spine motion at three cervical segments (occiput–C1, C1–C2, and C2–C5). We defined cervical spine motion as the change in angle between before and during tracheal intubation at each segment. Sagittal images were analyzed with a picture archiving and communication system (M-view, version 5.4; Infinitt Healthcare, Seoul, Korea) and reassessed by an anesthesiologist blinded to the study group assignment, to measure the amount of cervical spine movement. The angle was determine at the three cervical segments before and during tracheal intubation. Similar to previous studies, [4, 15, 16] we defined the reference line for the occiput segment as a line between the base of the sella and opisthion, and the C1 reference line as a line between the lower cortical margin of the anterior arch of C1 and the lower cortical margin of the C1 spinous process. We defined the C2 reference line as a line between the anterior, inferior margin of the C2 body and the lower cortical margin of the C2 spinous process. We defined the C5 reference line as a tangent along the superior endplate of the C5 body (Fig. 2).

The intubation time and number of intubation trials were noted. After extubation, the existence of bleeding in the oral cavity or a blood-tinged endotracheal tube was determined. In the post-anesthesia care unit, we evaluated the severity of sore throat using an 11-point (0–10) numerical rating scale.

### Statistical analysis

Linear mixed-effects models was used to compare the amount of cervical spine motion during intubation between the Macintosh laryngoscope first group and the C-MAC D-Blade videolaryngoscope first group and to determine a differential carryover effect between the two intubation sequences. For the primary outcome measure, the α value was adjusted to 0.0166 using the Bonferroni correction for multiple comparisons. Otherwise, a *p* value < 0.05 was considered to indicate statistical significance. R statistical software (ver. 3.4.3; R Foundation for Statistical Computing, Vienna, Austria) and SPSS 23.0 (SPSS Inc., Chicago, IL, USA) were used for the statistical analyses.

### Sample size determination

A previous study showed that the cervical spine motion at the occiput–C1 segment was 15 ± 7° during intubation with a Macintosh laryngoscope [4]. On the assumption that a 5 ± 7° reduction in cervical spine motion of the occiput–C1 segment is significant, the required sample size was calculated to be 18 patients (α = 0.05 and power = 0.80; two-sided comparison) with G*Power software (ver. 3.1.9.2; Franz Faul Universität Kiel, Germany). A total of 20 patients were needed to compensate for a predicted dropout rate of 10%.

## Results

Total 23 patients was eligible for the study, but 3 patients were excluded (Fig. 1). The remaining 20 patients were randomized to receive the allocated interventions. All patients were included in the data analysis and there were no dropouts. There were no significant differences in demographic or airway evaluation data between the Macintosh laryngoscope first and C-MAC D-Blade videolaryngoscope first groups (Table 1).

**Table 1:** Demographic and airway-related data

	Macintosh laryngoscope First(*n* = 10)	C-MAC D-Blade videolaryngoscope First(n = 10)
Age (yr)	59.1 ± 3.9	61.0 ± 11.6
Sex (male: female)	3: 7	2: 8
Height (cm)	159.6 ± 6.7	156.8 ± 9.4
Weight (kg)	61.6 ± 9.1	58.6 ± 9.7
Body mass index (kg/m^2^)	24.1 ± 2.5	23.8 ± 2.5
Mallampati score (1/2/3/4)	8/2/0/0	6/3/1/0
Inter-incisor gap (mm)	35.0 (34.0–43.5)	41.0 (36.8–49.0)
Thyromental distance (mm)	100.0 (90.0–105.0)	97.5 (87.5–111.3)
Neck circumference (cm)	35.0 (31.9–38.1)	33.5 (31.8–36.1)
Sternomandibular distance (mm)	152.5 (143.8–167.5)	155.0 (133.8–177.5)

Values are mean ± SD, median (IQR), or number

Our data showed that previous intubation had a nonsignificant carryover effect on the following intubation (*p* = 0.608, 0.451, and 0.388 for occiput–C1, C1–C2, and C2–C5, respectively). Regarding Macintosh laryngoscopic intubation, the mean difference (98.33% CI) in cervical spine motion between the second and first intubation was 3.0° (− 1.3 to 7.3), 2.6° (− 1.3 to 6.5), and − 1.4° (− 11.2 to 8.4) at occiput–C1, C1–C2, and C2–C5, respectively. Regarding C-MAC D-Blade videolaryngoscopic intubation, the mean difference (98.33% CI) in cervical spine motion between the second and first intubation was − 1.5° (− 7.0 to 4.0), − 0.8° (− 4.7 to 3.1), and 5.8 ° (− 1.4 to 13.0) at occiput–C1, C1–C2, and C2–C5, respectively.

**Fig. 3:**
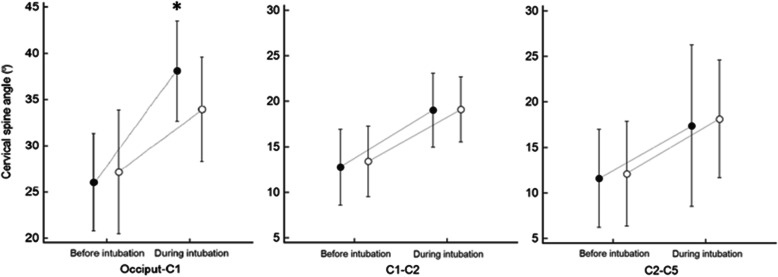
Cervical spine angles measured at three cervical segments before and during intubation with the C-MAC D-Blade videolaryngoscope (open circle) and the direct Macintosh laryngoscope (closed circle). Values are shown as mean ± SD. *: *p* < 0.05 vs. C-MAC D-Blade videolaryngoscope

The angles determined at each segment before intubation did not show statistically significant differences between the Macintosh laryngoscope and the C-MAC D-Blade videolaryngoscope. But, the cervical angle determined during intubation only at occiput–C1 segment was significantly larger with the Macintosh laryngoscope than the C-MAC D-Blade videolaryngoscope (38.1 ± 5.4°vs. 33.9 ± 5.7°; *p* = 0.022, Fig. 3).

The cervical spine motion of the occiput–C1 segment was 12.1 ± 4.2° and 6.8 ± 5.0° with the Macintosh laryngoscope and C-MAC D-Blade videolaryngoscope respectively, corresponding to a 44% reduction in cervical spine motion when the C-MAC D-Blade videolaryngoscope was used (mean difference [98.33% CI]; − 5.3 [− 8.8 to − 1.8]; *p* = 0.001; Table 2). At all other cervical segments, there was no significant difference between the two intubation devices (− 0.6 [− 3.4 to 2.2]; *p* = 0.639 in the C1–C2 segment and 0.2 [− 6.0 to 6.4]; *p* = 0.929 in the C2–C5 segment).

**Table 2:** Cervical spine motion at three cervical segments

	Macintosh laryngoscope (*n* = 20)	C-MAC D-Blade videolaryngoscope (n = 20)	Mean difference (98.33% CI)	*P* value
Occiput-C1 (°)	12.1 ± 4.2	6.8 ± 5.0	− 5.3 (− 8.8 to − 1.8)	0.001*
C1-C2 (°)	6.3 ± 3.8	5.7 ± 3.6	−0.6 (− 3.4 to 2.2)	0.639
C2-C5 (°)	5.8 ± 8.9	6.0 ± 7.3	0.2 (− 6.0 to 6.4)	0.929

Values are mean ± SD. CI, confidence interval. *: statistically significant after multiple comparisons (*P* < 0.05/3)

All patients were intubated successfully on the first attempt. There was no significant difference in intubation time between Macintosh laryngoscopic intubation and C-MAC D-Blade videolaryngoscopic intubation (6.1 [4.6–8.1] vs. 5.0 [3.4–9.5] s; *p* = 0.544). Sore throat was not significantly different between the Macintosh laryngoscope first and C-MAC D-Blade videolaryngoscope first groups (3.0 [0.8–7.0] vs. 3.5 [2.0–6.0]; *p* = 0.676). Oral cavity bleeding and a blood-tinged tracheal tube were observed in one patient in each group.

## Discussion

In this study, we compared cervical spine motion during tracheal intubation between the Macintosh laryngoscope and C-MAC D-Blade videolaryngoscope in patients with simulated cervical immobilization. The major finding was of significantly less cervical spine motion, with a 44% reduction at the occiput–C1 segment, during C-MAC D-Blade videolaryngoscopic intubation compared to Macintosh laryngoscopic intubation.

Maintaining cervical immobilization is essential during tracheal intubation in patients with an unstable cervical spine. This study showed superiority of the C-MAC D-Blade videolaryngoscope to the Macintosh laryngoscope, in terms of upper cervical spine motion, in patients with cervical immobilization. Macintosh laryngoscopy requires alignment of the three airway axes for visualization of the glottis, which is necessarily accompanied by cervical spine movement, especially extension of the upper cervical spine, even in cases with cervical immobilization. However, the C-MAC D-Blade videolaryngoscope provides an indirect view of the glottis without the requirement for strict alignment of the three airway axes. In addition, its acute angulation allows for reduced upper cervical spine extension during intubation.

In clinical practice, various videolaryngoscopes have been used for effective airway management in patients with difficult airways. However, there is controversy regarding cervical spine motion during tracheal intubation with direct laryngoscopes versus videolaryngoscopes. Similar to our study, a previous study demonstrated that the C-MAC D-Blade videolaryngoscope produced less cervical spine movement at the occiput–C1, occiput–C2, and occiput–C5 segments than the Macintosh laryngoscope [14]. Other studies showed that the GlideScope, Airway Scope, King Vision™ videolaryngoscope, Airtraq videolaryngoscope, and McGrath series 5 videolaryngoscope were also associated with less cervical spine motion during intubation, at various cervical segments, compared to the Macintosh laryngoscope [3–6, 17]. In contrast, other studies showed no significant difference in cervical spine motion between the direct laryngoscope and GlideScope in patients with manual in-line stabilization, or between the direct laryngoscope and Airtraq videolaryngoscope in a cadaver model of C1–C2 instability [15, 18]. A possible explanation for this discrepancy is that differences in the extent of airway muscle relaxation and neck immobilization, the experience of intubator, the shape and length of the videolaryngoscopic blade, and the force needed to elevate the videolaryngoscopic blade upward and forward can cause differences in cervical spine motion.

In this study, the average cervical spine motion was 6.8° at the occiput–C1 segment during C-MAC D-Blade videolaryngoscopic intubation. Similarly, a previous study reported cervical spine motion of 5.0° at the same cervical segment during C-MAC D-Blade videolaryngoscopic intubation [14]. These cervical spine motions at the occiput–C1 segment seem to be less during intubation with the C-MAC D-Blade videolaryngoscope compared to other videolaryngoscopes, as shown in studies in which the average cervical spine motion at the occiput–C1 segment was 9.1–12.5° and 10.4° for GlideScope and McGrath MAC videolaryngoscopic intubation, respectively [4, 15, 19]. These findings suggest superiority of the C-MAC D-Blade videolaryngoscope to other videolaryngoscopes in terms of upper cervical spine motion during intubation in cases with cervical immobilization. Differences in the shape and angle of the videolaryngoscope blade tip can explain why upper cervical spine motion is different between the C-MAC D-Blade videolaryngoscope and other videolaryngoscopes. The C-MAC D-Blade videolaryngoscope is characterized by greater curvature of the blade and a more acute angle of the blade tip than the McGrath MAC videolaryngoscope. Also, although the C-MAC D-Blade videolaryngoscope and the GlideScope have similar angulations, the former has a shorter angulated blade, which may affect the force needed to elevate the blade upward and forward for visualization of the glottis, resulting in a difference in cervical spine motion.

In this study, the average cervical spine motion at the C1-C2 segment was 6.3° during C-MAC D-Blade videolaryngoscopic intubation. Similar with our results, a previous study showed that the average cervical spine motion at the C1-C2 segment was 6.0° during McGrath MAC videolaryngoscopic intubation [19]. Also, another previous study showed a similar result on cervical spine motion at the C1-C2 segment (the median value: 6.0°) when the Airway Scope videolaryngoscope was used for intubation [3]. By contrast, a previous study conducted by Dr. Hindman and coworkers showed that the cervical spine motion at the C1-C2 segment was 2.3° during Airtraq videolaryngoscopic intubation [20]. Taken together, although the cervical spine motion at the C1-C2 segment depends on a videolaryngoscope used for intubation, it seems to be similar during intubation with various videolaryngoscopes.

In this study, the intubation time was not significantly different between C-MAC D-Blade videolaryngoscopic intubation and Macintosh laryngoscopic intubation. Also, the incidence of postoperative airway-related complications and the success rate at the first intubation attempt were comparable. However, caution is needed when interpreting the results because of the small sample size. A large scale prospective study is needed to compare clinical performance of intubation using both intubation devices. In general, cervical spine immobilization makes direct laryngoscopy difficult by hindering the alignment of three airway axes. Therefore, alternative intubation devices such as videolaryngoscopes and ETView Single Lumen laryngoscopes, and alternative intubation method (for example, blind intubation via a supraglottic airway device) have been introduced to increase the success rate of intubation in patients or manikins with cervical spine immobilization and showed their beneficial results on increased success rates of tracheal intubation [9, 10, 21–25].

In clinical practice, various neck collars have been used for cervical spine stabilization. They affect not only the extent of cervical spine mobility but also optic nerve sheath diameter. In this study, we used the Philadelphia neck collar, which was known to be more restrictive in cervical spine motion than other commercial neck collars (Aspen, Miami J, and Miami J with Occian back) in a previous study [26]. Its capability to restrict cervical spine motion during intubation was partially responsible for an increase in upper cervical spine motion shown in direct Macintosh laryngoscopy. Also, applying the neck collar affects optic nerve sheath diameter by obstructing venous drainage via compression of the neck veins. A recent clinical study showed a significant increase in the optic nerve sheath diameter from the baseline in healthy volunteers 5 and 20 min after applying the Philadelphia neck collar [27]. Therefore, great caution is needed when the neck collar is used for patients with increased intracranial pressure.

This study had several limitations. All intubations were performed by one anesthesiologist, who was not blinded to the intubating device; this may have led to a bias. A neck collar was applied for simulated cervical immobilization, although manual in-line stabilization of the neck is widely used in clinical practice for cervical immobilization. Further studies are needed to determine which method is better with regard to cervical spine motion during intubation. Although a sample size in this study is appropriate to perform the analyses, the relatively small sample size of the study group can limit generalizability. Also, when interpreting our results, possible differences in the anatomy of individuals including cervical spine mobility should be considered. We used a static image instead of a fluoroscopic image to avoid unnecessary exposure to radiation.

## Conclusions

This study demonstrated that cervical spine motion during tracheal intubation was significantly decreased when the C-MAC D-Blade videolaryngoscope was used with application of a neck collar for simulated cervical immobilization. This result suggests that use of the C-MAC D-Blade videolaryngoscope may be beneficial in patients with an unstable cervical spine, in terms of cervical spine motion during tracheal intubation.

## Data Availability

The datasets used and/or analyzed during the current study are available from the corresponding author on reasonable request.

## Abbreviation

CONSORT: Consolidated Standards of Reporting Trials
